# NAD^+^ is critical for maintaining acetyl-CoA and H3K27ac in embryonic stem cells by Sirt1-dependent deacetylation of AceCS1

**DOI:** 10.1093/lifemedi/lnac046

**Published:** 2022-10-26

**Authors:** Yi Wu, Yang Liu, Zhihong Hao, Xingguo Liu

**Affiliations:** CAS Key Laboratory of Regenerative Biology, Joint School of Life Sciences, Guangzhou Institutes of Biomedicine and Health, Chinese Academy of Sciences, Guangzhou Medical University, Guangzhou 510530, China; Guangdong Provincial Key Laboratory of Stem Cell and Regenerative Medicine, China-New Zealand Joint Laboratory on Biomedicine and Health, CUHK-GIBH Joint Research Laboratory on Stem Cells and Regenerative Medicine, Institute for Stem Cell and Regeneration, Guangzhou Institutes of Biomedicine and Health, Chinese Academy of Sciences, Guangzhou 510530, China; CAS Key Laboratory of Regenerative Biology, Joint School of Life Sciences, Guangzhou Institutes of Biomedicine and Health, Chinese Academy of Sciences, Guangzhou Medical University, Guangzhou 510530, China; Guangdong Provincial Key Laboratory of Stem Cell and Regenerative Medicine, China-New Zealand Joint Laboratory on Biomedicine and Health, CUHK-GIBH Joint Research Laboratory on Stem Cells and Regenerative Medicine, Institute for Stem Cell and Regeneration, Guangzhou Institutes of Biomedicine and Health, Chinese Academy of Sciences, Guangzhou 510530, China; University of Chinese Academy of Sciences, Beijing 100049, China; CAS Key Laboratory of Regenerative Biology, Joint School of Life Sciences, Guangzhou Institutes of Biomedicine and Health, Chinese Academy of Sciences, Guangzhou Medical University, Guangzhou 510530, China; Guangdong Provincial Key Laboratory of Stem Cell and Regenerative Medicine, China-New Zealand Joint Laboratory on Biomedicine and Health, CUHK-GIBH Joint Research Laboratory on Stem Cells and Regenerative Medicine, Institute for Stem Cell and Regeneration, Guangzhou Institutes of Biomedicine and Health, Chinese Academy of Sciences, Guangzhou 510530, China; University of Chinese Academy of Sciences, Beijing 100049, China; CAS Key Laboratory of Regenerative Biology, Joint School of Life Sciences, Guangzhou Institutes of Biomedicine and Health, Chinese Academy of Sciences, Guangzhou Medical University, Guangzhou 510530, China; Guangdong Provincial Key Laboratory of Stem Cell and Regenerative Medicine, China-New Zealand Joint Laboratory on Biomedicine and Health, CUHK-GIBH Joint Research Laboratory on Stem Cells and Regenerative Medicine, Institute for Stem Cell and Regeneration, Guangzhou Institutes of Biomedicine and Health, Chinese Academy of Sciences, Guangzhou 510530, China; Centre for Regenerative Medicine and Health, Hong Kong Institute of Science & Innovation, Chinese Academy of Sciences, Hong Kong SAR, China

**Dear Editor,**

Cell fate determination coincides with profound organelle remodeling and metabolic switch, and metabolites are emerging as critical regulators of epigenetics and cell fate transitions [1]. The functional roles and mechanisms of a single metabolite on cell fate determination have been extensively studied [2]. However, how different metabolites are interlinked in cell fate transitions has been poorly explored. We recently reported that lipid droplet mobilization accelerates the differentiation of mouse embryonic stem cells (mESCs) [3]. Loss of lipid droplet protein Plin2 promotes lipid hydrolysis from lipid droplets and remodels lipidome, resulting in disorganized mitochondrial cristae and decreased mitochondrial activity. The impaired mitochondrial activity reduces acetyl-CoA generated from mitochondrial fatty acid oxidation (FAO), which ultimately leads to reduced histone acetylation and accelerated pluripotency exit of mESCs. Notably, we also observed a significant decrease in nicotinamide adenine dinucleotide (NAD^+^) level in Plin2 knockout (Plin2^−/−^) mESCs. NAD^+^ is a coenzyme central to cellular metabolism and closely linked to mitochondria activity [4], which may contribute to acetyl-CoA-mediated cell fate transition in mESCs.

To investigate whether NAD^+^ plays a role in the regulation of acetyl-CoA and pluripotency in mESCs, we measured NAD^+^/NADH ratio and confirmed NAD^+^/NADH was decreased in undifferentiated and differentiating Plin2^−/−^ mESCs compared with that in wild type (WT) mESCs, suggesting the NAD^+^ decline results in reduced NAD^+^/NADH ratio in mESCs (Fig. 1A). To investigate the role of NAD^+^ in Plin2-mediated differentiation of mESCs, we tested the effects of nicotinamide mononucleotide (NMN), a precursor of NAD^+^, in differentiation [5]. We included acetate, which could be converted to acetyl-CoA and delay differentiation of ESC [6], as a positive control. Intriguingly, NMN could delay the differentiation of Plin2^−/−^ mESCs, while acetate could not, indicated by the percentage of Oct4 positive cells and expression of pluripotency associated genes (Fig. 1B and 1C). We further confirmed that the NAD^+^/NADH ratio was increased by NMN treatment in Plin2^−/−^ mESCs (Fig. 1D). In addition, Plin2^−/−^ mESCs treated with NMN exhibited a dome-shaped morphology during early stage of differentiation, and was able to differentiate into all three germ layers at late stage of differentiation (Fig. S1A and S1B). Because reduced acetyl-CoA is responsible for Plin2-mediated differentiation, we examined acetyl-CoA levels in WT and Plin2^−/−^ mESCs in the presence of NMN or acetate. We found that acetate could not increase acetyl-CoA levels in Plin2^−/−^ mESCs, whereas NMN treatment increased acetyl-CoA significantly (Fig. 1E). Importantly, supplementation of NMN increased acetylation of histone H3 at lysine 27 (H3K27ac) (Fig. 1F). Taken together, these results demonstrate that increasing NAD^+^ level by supplementation of NAD^+^ precursor NMN could increase acetyl-CoA and histone acetylation, and delay the differentiation of Plin2^−/−^ mESCs.

**Figure 1.**
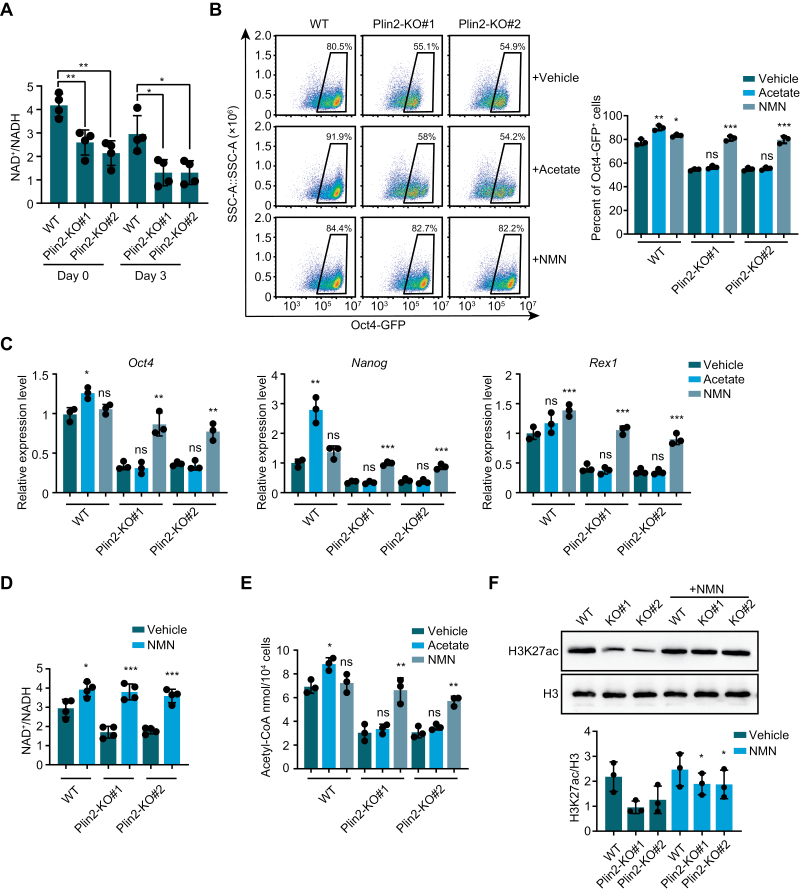
NAD^+^ precursor NMN delays differentiation by increasing acetyl-CoA and H3K27ac level. (A) NAD^+^/NADH in WT and Plin2^−/−^ mESCs (KO#1 and KO#2) on day 0 and day 3 of differentiation. Data are mean ± SD, *n* = 4 biological replicates. Two-tailed unpaired *t*-tests. **P < 0.005, *P < 0.05. (B) Flow cytometry analysis and quantification of Oct4-GFP^+^ cells in WT and Plin2^−/−^ mESCs (KO#1 and KO#2) on day 3 of differentiation in the presence of acetate or NMN. Data are mean ± SD, *n* = 3 biological replicates. Two-tailed unpaired *t*-tests. ***P < 0.001, **P < 0.005, *P < 0.05. (C) qRT-PCR analysis of expression of pluripotency genes (*Oct4*, *Nanog*, and *Rex1*) in WT and Plin2^−/−^ mESCs (KO#1 and KO#2) on day 3 of differentiation in the presence of acetate or NMN. Data are mean ± SD, *n* = 3 biological replicates. Two-tailed unpaired *t*-tests. ***P < 0.001, **P < 0.005, *P < 0.05. (D) NAD^+^/NADH in WT and Plin2^−/−^ mESCs (KO#1 and KO#2) treated with vehicle or NMN. Data are mean ± SD, *n* = 4 biological replicates. Two-tailed unpaired *t*-tests. ***P < 0.001, *P < 0.05. (E) Acetyl-CoA level in WT and Plin2^−/−^ mESCs (KO#1 and KO#2) treated with vehicle or NMN. Data are mean ± SD, *n* = 3 biological replicates. Two-tailed unpaired *t*-tests. **P < 0.005, *P < 0.05. (F) Up: Western blot analysis of histone acetylation (H3K27ac) in WT and Plin2^−/−^ mESCs (KO#1 and KO#2) treated with vehicle or NMN. Representative blot is shown. Down: Quantification of relative H3K27ac. Data are mean ± SD, *n* = 3 biological replicates. Two-tailed unpaired *t*-tests. *P < 0.05.

Because disorganized mitochondrial cristae and decreased FAO ability of mitochondria are responsible for the differentiation of Plin2 knockout mESCs [3], we ask whether NMN supplementation increases cellular acetyl-CoA by restoring mitochondrial cristae structure and FAO. Transmission electron microscope analysis showed disorganized mitochondrial cristae in Plin2^−/−^ mESCs treated with NMN, suggesting that NAD^+^ could not restore mitochondrial cristae (Fig. 2A). In addition, we observed a slight change in mitochondrial size upon NMN treatment. However, supplementation of NMN resulted in a remarkable increase in cellular oxidation of both exogenous and endogenous fatty acids (Fig. 2B), indicating the increase in acetyl-CoA might be due to increased FAO. We hypothesized whether increased cellular FAO is caused by changes in mitochondrial numbers. We therefore measured mitochondrial amount by analysis of mitochondrial protein Tom20 and mtDNA levels in Plin2^−/−^ mESCs treated with NMN. Supplementation of NMN significantly increased Tom20 protein levels and mtDNA levels in Plin2^−/−^ mESCs (Fig. 2C and 2D), suggesting that NAD^+^ increases cellular FAO and acetyl-CoA production by increasing the number of mitochondria in mESCs, but not likely affect FAO ability of mitochondria.

**Figure 2.**
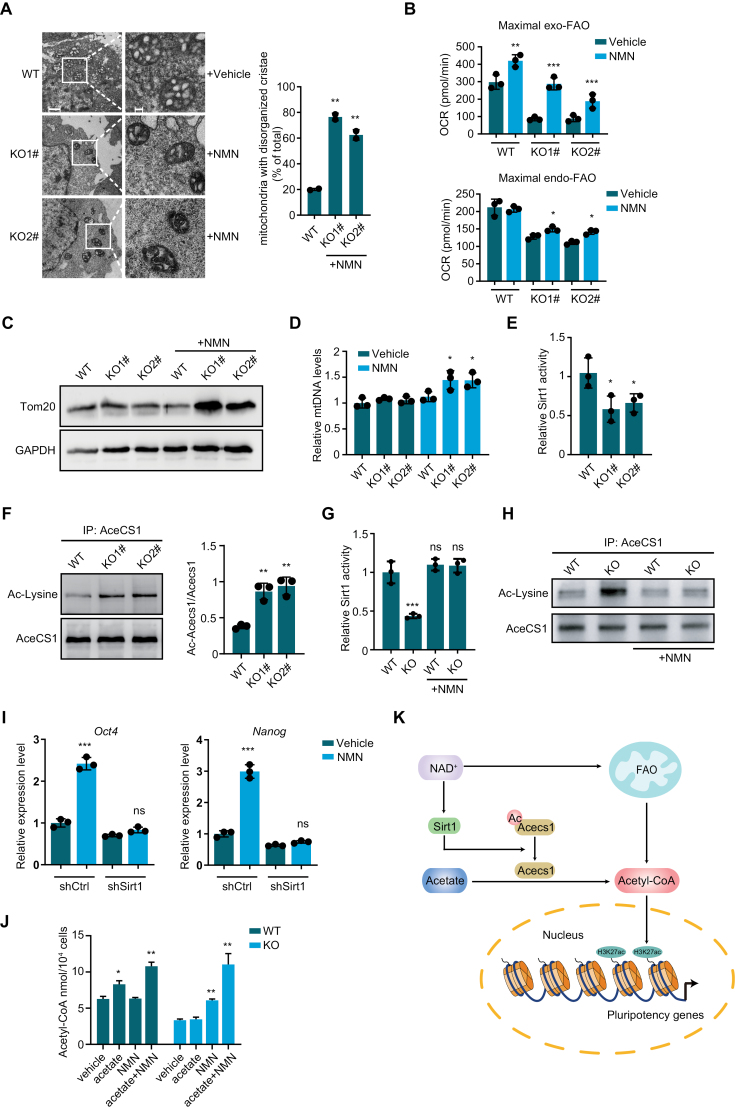
NAD^+^ is critical for the conversion of acetate to acetyl-coA by increasing Sirt1-dependent deacetylation of Acecs1 in ESCs. (A) Representative TEM images of mitochondria and quantification of mitochondria with disorganized cristae in WT and Plin2^−/−^ mESCs (KO1# and KO2#) treated with vehicle or NMN. Scale bar, left: 1 μm, right: 200 nm. Data are mean ± SD, *n* = 2 biological replicates (each >50 mitochondria). two-tailed unpaired *t*-tests. **P < 0.005. (B) Seahorse analysis of maximal endogenous and exogenous FAO in WT and Plin2^−/−^ mESCs (KO1# and KO2#) treated with vehicle or NMN. Data are mean ± SEM, *n* = 3 biological replicates. two-tailed unpaired *t*-tests. ***P < 0.001, **P < 0.005, *P < 0.05. (C) Western blot analysis of Tom20 in WT and Plin2^−/−^ mESCs (KO1# and KO2#) treated with vehicle or NMN. The experiments were repeated independently twice with similar results. (D) qPCR analysis of mtDNA copy numbers in WT and Plin2^−/−^ mESCs (KO1# and KO2#) treated with vehicle or NMN. Data are mean ± SD, *n* = 3 biological replicates. *P < 0.05. (E) Relative Sirt1 deacetylase activity in WT and Plin2^−/−^ mESCs (KO1# and KO2#). Data are mean ± SD, *n* = 3 biological replicates. *P < 0.05. (F) Western blot analysis of acetylation of AceCS1 in WT and Plin2^−/−^ mESCs (KO1# and KO2#). Data are mean ± SD, *n* = 3 biological replicates. **P < 0.005. (G) Relative Sirt1 deacetylase activity in WT and Plin2^−/−^ mESCs treated with vehicle or NMN. Data are mean ± SD, *n* = 3 biological replicates from KO1# and KO2#. ***P < 0.001, ns: none significant. (H) Western blot analysis of acetylation of AceCS1 in WT and Plin2^−/−^ mESCs treated with vehicle or NMN. The experiments were repeated independently twice with similar results. (I) qRT-PCR analysis of expression of pluripotency genes (*Oct4* and *Nanog*) in Plin2^−/−^ mESCs transfected with shRNA against Sirt1 on day 3 of differentiation in the presence of vehicle or NMN. Data are mean ± SD, *n* = 3 biological replicates. Two-tailed unpaired *t*-tests. ***P < .001, ns: none significant. (J) Acetyl-CoA levels in WT and Plin2^−/−^ mESCs in the presence of acetate, NMN or acetate plus NMN. Data are mean ± SD, *n* = 2 biological replicates from KO1# and KO2#. **P < 0.005, *P < 0.05. (K) Graphical summary.

Next, we sought to clarify why acetate treatment could not replenish cellular acetyl-CoA pools in Plin2^−/−^ mESCs. Acetate can be converted to acetyl-CoA by acetyl-CoA synthase 1 (AceCS1), whose enzymatic activity is enhanced by sirtuin 1 (Sirt1)-mediated deacetylation [7]. Because NAD^+^ level is critical for Sirt1 activity and significantly reduced in Plin2^−/−^ mESCs [8], we reasoned that decreased Sirt1 activity and increased acetylation of AceCS1 might be responsible for the failure of acetate to restore acetyl-CoA levels. To address this, we measured Sirt1 deacetylase activity and found Sirt1 activity was decreased in Plin2^−/−^ mESCs (Fig. 2E). Next, we analyzed the acetylation status of AceCS1 by immunoprecipitation of AceCS1 and immunoblot of lysine acetylation. The results showed that the acetylation of AceCS1 was increased in Plin2^−/−^ mESCs, compared with WT mESCs (Fig. 2F). We further tested the effects of NMN treatment on Sirt1 activity and AceCS1 acetylation in WT and Plin2^−/−^ mESCs. NMN treatment greatly increased Sirt1 activity and decreased AceCS1 acetylation in Plin2^−/−^ mESCs, while had little effects in WT mESCs (Fig. 2G and 2H). Moreover, NMN could not delay differentiation when Sirt1 expression was knocked down (Fig. 2I), indicating Sirt1 may be critical for the effects of NMN on pluripotency. These results also indicate that Sirt1-mediated deacetylation of AceCS1 is critical for the conversion of acetate to acetyl-CoA by AceCS1, promoting us to ask whether acetate could be converted to acetyl-CoA when NAD^+^ level is restored. We therefore tested the effects of acetate in the presence of NAD^+^ precursor NMN. Although acetate alone did not increase acetyl-CoA levels in Plin2^−/−^ mESCs, it increased acetyl-CoA levels in WT mESCs, which exhibited higher NAD^+^ levels (Fig. 2J). Importantly, supplementation of acetate in the presence of NMN resulted in a significantly increase in acetyl-CoA levels in both WT and Plin2^−/−^ mESCs (Fig. 2J). Altogether, these results suggest NAD^+^ level is critical for AceCS1-mediated generation of acetyl-CoA in mESCs.

In this study, we show NAD^+^ is critical for acetyl-CoA and histone acetylation in mESCs, and elucidated the interplay between NAD^+^ and acetyl-CoA. Increasing NAD^+^ by supplementation of NMN increases mitochondrial amount, leading to increased mitochondrial FAO and acetyl-CoA production. On the other hand, NAD^+^ improves Sirt1 deacetylase activity and reduced acetylation of AceCS1, thus increasing AceCS1-mediated conversion of acetate to acetyl-CoA (Fig. 2H). Our findings reveal NAD^+^ orchestrates histone acetylation by regulating Sirt1 activity and acetyl-CoA availability. Given that NAD^+^ decline is associated with various physiological and pathological processes [9], our discovery linking NAD^+^ to acetyl-CoA and epigenetic modifications will shed light on the mechanisms underlying development and diseases.

## Research limitations

Our results implicate that the NAD^+^-Sirt1 axis is critical for acetyl-CoA and histone acetylation in mESCs. How this NAD^+^-Sirt1 axis regulates mitochondrial size and numbers is still unclear. Our findings linking NAD^+^ to acetyl-CoA and epigenetic modifications should also play roles in mammalian development and human ESCs. Much work is needed to uncover its roles in mammalian development and human ESCs.

## Supplementary Material

lnac046_suppl_Supplementary_Material
