# Supplementary material for: NAD+ is critical for maintaining acetyl-CoA and H3K27ac in embryonic stem cells by Sirt1-dependent deacetylation of AceCS1
Source: Life Med. 2022 Oct 26;1(3):401–5. doi: 10.1093/lifemedi/lnac046 (PMC11749344; doi:10.1093/lifemedi/lnac046)
Supplement: lnac046_suppl_Supplementary_Material [file lnac046_suppl_Supplementary_Material.pdf]

Supplementary Information for "NAD^+ is critical for maintaining acetyl-CoA and H3K27ac in embryonic stem cells by Sirt1-dependent deacetylation of AceCS1"

Materials and Methods

Cell culture

OG2 mESCs were derived from E3.5 embryos carrying the *Oct4*-GFP transgenic allele and cultured on 0.1% gelatin-coated plates in DMEM/high glucose supplemented with N2 (Gibco, 200×), B27 (Gibco, 100×), sodium pyruvate (Gibco, 100×), GlutaMAX (Gibco, 100×), NEAA (Gibco, 100×), 0.1 mM 2-mercaptoethanol (Gibco), 1,000 U/mL leukemia inhibitory factor (LIF), 3 μM CHIR99021 (Selleck) and 1 μM PD0325901 (Selleck). The cells were obtained with approval from the ethics committee of the Guangzhou Institutes of Biomedicine and Health, Chinese Academy of Sciences (GIBH). All the animals were handled according to approved Institutional Animal Care and Use Committee (IACUC) protocols of the GIBH.

Plasmids

shRNA inserts (shSirt1) were cloned into pLKO.1 lentiviral vector. The shRNA target sequences are AGTGAGACCAGTAGCACTAAT and GCCATGTTTGATATTGAGTAT.

Spontaneous differentiation of mESCs

To induce spontaneous differentiation, mESCs were dissociated into single cells using 0.25% trypsin-EDTA (Gibco). The cell suspension (6×10^5 cells/ml) was plated in 100 mm non-adherent dish and cultured in Knockout DMEM (Gibco) supplemented with 15% KSR (Gibco), GlutaMAX (Gibco, 100×) and NEAA (Gibco, 100×) for 3 days. After 3 days, they were plated in gelatin-coated dish and cultured for indicated days.

Quantitative reverse transcription PCR (qRT-PCR)

Total RNA was extracted by TRIzol (Invitrogen) and 5 μg RNA was used to

generate complementary DNA (cDNA). cDNA levels were determined by ChamQ SYBR qPCR Master Mix (Vazyme Biotech Co.,Ltd) and analyzed with a CFX-96 Real-Time system (Bio-Rad). The primers used are from our previous reports (Wu et al., 2022).

Immunoprecipitation and western blot

Whole cell extracts were obtained with cell lysis buffer (50 mM Tris-HCl pH7.4, 150 mM NaCl, 1mM EDTA, 1% NP-40 and protease inhibitor cocktail) for 30 min at 4°C. Anti-AceCS1 (Cell Signaling, 3658, 1:200) was linked to Dynabeads with protein A and G (Invitrogen) for 4 hours at 4°C in lysis buffer. Lysates were incubated with the washed antibody-linked Dynabeads overnight at 4°C. After immunoprecipitation, beads were washed with lysis buffer five times and boiled in SDS buffer for 10 min. The eluents were analyzed by western blot. The following primary antibodies were used: anti-AceCS1 (Cell Signaling, 3658, 1:1000), anti-acetylated-Lysine (Cell Signaling, 9441, 1:500), anti-GAPDH (Bioworld, AP2063, 1:5000), anti-H3K27ac (Cell Signaling, 8173, 1:1000), anti-histone H3 (Abcam, ab1791, 1:5000) and anti-Tom20 (Abcam, ab56783, 1:200). The secondary antibodies were goat anti-rabbit (Kangchen, KC-RB-035) and goat anti-mouse (Kangchen, KC-MM-035).

Flow cytometry

For analysis of Oct4-GFP$^+$ cells, cells were washed with PBS once and dissociated into single cell with trypsin-EDTA (0.25%). The cells were analyzed with an Accuri C6 flow cytometer (BD Biosciences).

Transmission Electron Microscope (TEM)

Cells were washed with PBS and fixed with 3% glutaraldehyde (Sigma) for 12 hours at 4 °C. Next, cells were fixed with 1% osmium acid (SPI-Chem) for another 1 hour before dehydration by gradient ethanol. After fixation, the samples were stained with lead (SPI-Chem) and uranium (SPI-Chem). After ultra-sectioning with Leica EM UC7, the ultrastructure of mitochondria was visualized by TEM (FEI Tecnai G2 Spirit, USA) at 120 keV.

Cellular acetyl-CoA and NAD$^+$/NADH measurement

Cellular acetyl-CoA and NAD^+/NADH were measured with the PicoProbe Acetyl-CoA fluorometric assay kit (Biovision, K317) and the NAD^+/NADH quantification colorimetric kit (Biovision, K337), respectively. For acetyl-CoA measurement, cells were washed with cold PBS and deproteinized using perchloric acid/KOH following the manufacturer's instruction. The samples were diluted with assay buffer, and incubated in CoA quencher for 5 min at room temperature. The fluorescence signal was measured at Excitation/Emission=535/587 nm. For NAD^+/NADH measurement, cells were washed with cold PBS and extracted with NADH/NAD extraction buffer by freeze/thaw two cycles. The extracted supernatant was incubated at 60 °C for 30 min to decompose NAD for detecting NADH. The signal was measured at OD 450 nm.

Fatty acid oxidation assay

Fatty acid oxidation (FAO) was analyzed using the XF24 extracellular flux analyzer (Seahorse Biosciences). A total of 1×10^6 cells were plated on gelatin coated plates overnight at 37°C. FAO were determined with the Seahorse XF palmitate-BSA FAO Substrate from the Seahorse XF Palmitate Oxidation Stress Kit according to the manufacturer's instructions. For FAO measurement, 2 μM oligomycin, 1 μM FCCP, 1 μM rotenone, 1 μM antimycin and 100 μM Etomoxir were used. Maximal exogenous FAO was determined by the increase of OCR after palmitate treatment in a substrate-limited assay medium which was derived of oxidation substrate such as glucose, pyruvate, and glutamine. Maximal endogenous FAO was determined by the increase of OCR after Eto treatment in a substrate-limited assay medium.

Statistical analysis

Data are presented as mean ± SD or mean ± SEM. Sample number (*n*) indicates the number of independent biological samples in each experiment. Statistical comparisons were performed using the unpaired two-tailed Student's *t* tests with GraphPad Prism 8. P value < 0.05 was considered statistically significant.

References

Wu, Y., Chen, K., Li, L., Hao, Z., Wang, T., Liu, Y., Xing, G., Liu, Z., Li, H., Yuan, H., *et al.* (2022). Plin2-mediated lipid droplet mobilization accelerates exit from pluripotency by lipidomic remodeling and

histone acetylation. Cell death and differentiation.

Figure S1

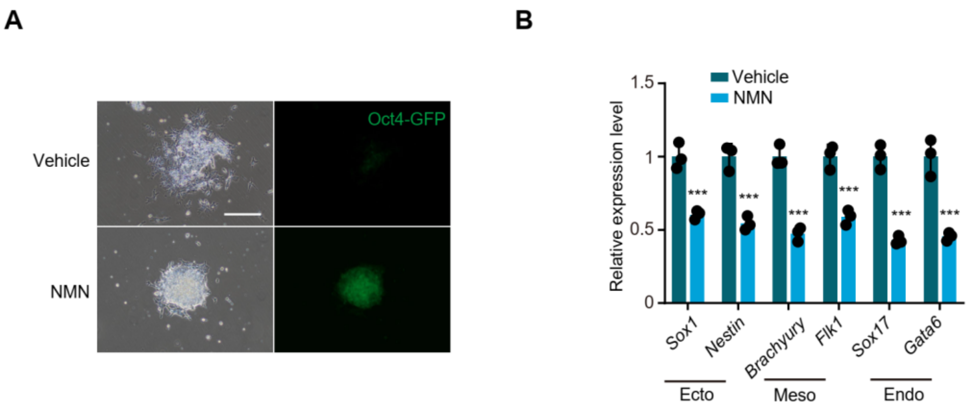

Figure S1. **NMN delays differentiation of Plin2$^{-/-}$ mESCs.**

(A) Representative phase-contrast and Oct4-GFP images of Plin2$^{-/-}$ mESCs on day 3 of differentiation in the presence of Vehicle or NMN.

(B) qRT-PCR analysis of markers for ectoderm (*Sox1*, *Nestin*), mesoderm (*Brachyury*, *Flk1*) and endoderm (*Sox17*, *Gata6*) in Plin2$^{-/-}$ mESCs on day 8 of differentiation in the presence of Vehicle or NMN. Data are mean ±s.d., n = 3 biological replicates. two-tailed unpaired t-tests. ***P < 0.001.
